# From Childhood to Old Age: Current Knowledge and Practical Approaches to Metabolic Dysfunction-Associated Steatotic Liver Disease

**DOI:** 10.3390/jcm15041536

**Published:** 2026-02-15

**Authors:** Iwona Gorczyca-Głowacka, Michał Tarnowski, Anna Zmelonek-Znamirowska, Przemysław Wolak

**Affiliations:** 1 Collegium Medicum, The Jan Kochanowski University, 25-317 Kielce, Poland; przemyslaw.wolak@ujk.edu.pl; 2 Department of Cardiology, Holy Spirit Specialist Hospital, 27-600 Sandomierz, Poland; michal.tarnowski8@gmail.com; 3 Clinic of Obstetrics and Gynecology, Provincial Combined Hospital, 25-736 Kielce, Poland; rjawka@poczta.fm

**Keywords:** childhood, elderly, metabolic dysfunction-associated steatotic liver disease, obesity, overweight

## Abstract

**Background**: Metabolic dysfunction-associated steatotic liver disease (MASLD) is one of the most common chronic liver diseases across all age groups. **Methods**: This review synthesizes the current evidence from landmark studies on the risk factors, diagnosis, and management of MASLD in pediatric, adult, and particularly elderly patients. **Results**: Based on the current data, we demonstrated that the prevalence of MASLD increases with age from childhood to middle adulthood, whereas, in elderly individuals, there is no further age-related increase observed. In the pathogenesis of the disease, familial and prenatal factors predominate in the youngest patients, while metabolic factors are the main contributors in adults. However, obesity remains the most significant risk factor for MASLD across all age groups. Therefore, systematic screening for MASLD should be strongly recommended in individuals with obesity. Laboratory parameters indicating an increased risk of MASLD are primarily recommended in screening regimens for children and adults; however, in elderly patients, these parameters may remain within normal ranges due to the long-standing disease course and progression toward fibrosis. On the basis of current studies and guidelines, we showed that lifestyle modification, including dietary changes and increased physical activity, is the cornerstone of treatment across all age groups. Nevertheless, non-pharmacological interventions have limitations in pediatric and elderly populations and are implemented less effectively in these groups than in middle-aged patients. **Conclusions**: The early identification of high-risk patients and implementation of multidisciplinary, age-targeted metabolic prevention strategies are essential to prevent MASLD progression and its non-liver complications.

## 1. Introduction

Over the past few years, metabolic dysfunction-associated steatotic liver disease (MASLD) has emerged as an increasingly significant health concern in people of all ages. MASLD is recognized as the most common cause of chronic liver disease in both children and adults, with the most recent meta-analyses suggesting that more than 38% of the world’s adult population and between 7% and 14% of children are affected [1,2].

Fatty liver disease was initially documented in the 19th century by Addison, though the notion of non-alcoholic steatohepatitis (NASH) emerged only in 1980 to characterize its progressive variant—which mirrored the histology of alcohol-induced steatohepatitis yet occurred in individuals reporting no alcohol consumption. The term was applied to pediatric cases in 1983, and non-alcoholic fatty liver disease (NAFLD) was coined in 1986 [3].

In 2020, an international panel of experts reclassified metabolically driven fatty liver disease and proposed metabolic-associated fatty liver disease (MAFLD) to supplant NAFLD [4]. In 2023, leading global hepatology organizations issued a unified consensus adopting MASLD as the new standard term [5]. This designation replaces both prior terms—NAFLD and MAFLD—by highlighting the metabolic foundations of the condition and eliminating the alcohol-related stigma, along with the derogatory phrase “fatty liver”. The change from MAFLD to MASLD nomenclature seeks to unify terminology across steatotic liver diseases in all age groups. This transition holds key ramifications, prioritizing metabolic dysfunction while accommodating established diagnostic processes and epidemiological evidence rooted in MASLD criteria [6,7,8]. Such redefinition more accurately captures the disease’s metabolic essence and offers substantial benefits for timely detection and management, especially among children.

MASLD arises from the interplay of metabolic, hormonal, genetic, and environmental factors. Its pathogenesis is complex, with MASLD patients commonly exhibiting metabolic syndrome elements, such as hyperlipidemia, type 2 diabetes mellitus (T2DM), obesity, and hypertension—earning it the label of hepatic manifestation of metabolic syndrome [9,10]. The disease’s worldwide surge and healthcare strain have followed a troubling, swift escalation in obesity and metabolic conditions [11,12]. Across different age groups, various factors predominate in the pathogenesis of MASLD.

Despite the numerous studies conducted on MASLD in various age demographics, a comprehensive review that specifically addresses MASLD in pediatric, adult, and older patients within the context of differences in epidemiology, diagnosis, and treatment is lacking. The present narrative review aims to emphasize the increasing prevalence of MASLD and synthesize current evidence on diagnostic criteria and therapeutic recommendations relevant to MASLD across all age groups.

## 2. Material and Methods

A narrative literature review was conducted using the PubMed, Embase, and Cochrane Library databases. It is acknowledged that the MASLD terminology was only established in 2023, and as such the extant literature on MASLD remains limited. Consequently, the data presented herein are based on historical nomenclature and diagnostic criteria for NAFLD, MAFLD, MASLD, and SLD. The search strategy employed a broad set of keywords and their synonyms. Search terms included “MASLD” or “MAFLD” or “NAFLD” or “SLD” and “pediatric” or “children” or “adolescent” or “adults” or “elderly” and “epidemiology” or “pathogenesis” or “treatment” or “management”. The review concentrated on studies involving the pediatric population, defined as children and adolescents up to the age of 18 years, the adult population, and the elderly population with MASLD. Studies conducted exclusively in animal models were excluded from the present analysis unless they provided relevant pathophysiological or translational insights. The publications issued between 2020 and 2025 were accorded priority in order to capture the most up-to-date evidence, emerging terminology and revised clinical practice guidelines. These publications were supplemented by earlier studies of notable clinical or scientific significance. The selection of studies followed a narrative approach grounded in clinical relevance, prioritizing randomized controlled trials, meta-analyses, and systematic reviews. In the present review, articles written in languages other than English were excluded from consideration.

Artificial intelligence tools were not used in the preparation of this manuscript.

## 3. Results

### 3.1. Epidemiology

MASLD has shown a steady increase over the past decade, reflecting shifts in dietary habits, reduced physical activity, and rising childhood obesity. This trend is observed across all age groups; however, it is less pronounced among the elderly compared to younger populations. Global trend analyses indicate that the prevalence of pediatric MASLD nearly doubled from 4.6% in 2000 to 9.0% in 2017, with epidemiological projections suggesting that rates may reach approximately 30.7% by 2040 [13]. It should be noted that epidemiological data may vary, primarily due to changing criteria for diagnosing the disease in recent years, and secondly due to the different assessments of patients—from clinical features to liver biopsy findings. Imaging diagnostics may also provide different diagnoses, depending on whether they are based on abdominal ultrasound, computed tomography, or magnetic resonance imaging.

A meta-analysis, which included studies conducted before 2013, estimated the global prevalence of MASLD in the general pediatric population at 7.6% [14]. However, a more recent meta-analysis published in 2024, including studies from 1997 to 2023, reported a global prevalence of 13% [15], indicating a rise in pediatric MASLD prevalence. It is important to note that obese and overweight children show markedly higher MASLD than the general pediatric population. The risk varies according to body mass index (BMI) categories, with prevalence rates of 12% in children with normal weight, 21% in those with overweight, and 47% in those with obesity [15]. A separate meta-analysis of 29 observational studies found that MASLD prevalence among obese and overweight adolescents and children was 33.8% (95%CI: 27.3–40.9%) [16]. Pediatric MASLD is detectable even in preschool-age children, with one birth cohort study in Shanghai reporting a prevalence of 0.5% at age 5, increasing to 3.4% by age 8 [17]. These findings indicate that disease may develop much earlier than once thought, highlighting the importance of preventive strategies in early life. Sex-related differences influence MASLD risk. Studies have reported a higher prevalence of MASLD in males compared with females within the pediatric population. A separate cross-sectional study indicated an overall MASLD prevalence of 18.5%, rising to 12% in girls and 24.5% in boys (OR 2.4; 95% CI: 1.1–5.2) [18].

The most recent meta-analysis revealed that 38% of adults worldwide had MASLD during 2016–2019, representing a 50% increase compared with 1990–2006 [1]. MASLD prevalence shows a substantial geographic variation, highlighting environmental influences on the disease. It peaks in Latin America at 44.4% and is lowest in Western Europe at 25.1% [1]. These patterns are projected to persist, with the world prevalence expected to reach 55.4% by 2040 [19]. Xu B et al. [20] analyzed prevalence, incidence, mortality, and disability-adjusted life years among individuals aged 15–49 years using data from the Global Burden of Disease 2021 study. Between 1990 and 2021, the number of individuals with MASLD increased from 343 million to 666 million, reflecting an average annual growth rate of 0.95%. Regarding sex-based differences, males exhibited higher rates of MASLD-related deaths and disability-adjusted life years compared to females, although prevalence increases were more pronounced among females. Age-group analysis revealed that the 45–49-year group experienced the most significant surge in MASLD burden. Among adults, MASLD also occurs more frequently in men. Data from the Polish Gallstone Surgery Registry revealed sex-specific differences in MASLD prevalence, with men showing rates of 29.8% compared with 21.9% in women, suggesting a greater male susceptibility to MASLD [21]. A Spanish study reported an overall MASLD prevalence of 19.1%, with rates of 27.9% in men and 6.8% in women, increasing across age groups [22]. In addition, the research by Chang et al. [23] indicated higher MASLD rates in males, with a percentage of 45.7%, as opposed to 23.9% in females. MASLD is also more prevalent in Asian populations [24,25]. The increased prevalence observed in the male population may be a contributing factor to their heightened vulnerability to chronic diseases, including cardiovascular disease and T2DM.

In contrast to other age groups, the burden of MASLD in the elderly is still inadequately characterized. While the pivotal Rotterdam research indicated a drop in MASLD prevalence as age increased, NHANES III results demonstrated mostly steady MASLD rates between those aged 60–74 years and those aged >74 years [26,27]. In addition, multiple markers of metabolic dysregulation correlate with MASLD across middle-aged and elderly populations [27,28]. Among 9847 Australian participants, the prevalence of MASLD was 33.0%, with a noted decrease from 34.8% in the 70–72-year group to just 21.1% in those 85 years or older. Males exhibited a greater MASLD prevalence (37.8%) than females (29.0%), yet this age-driven reduction appeared in both genders. The age- and sex-standardized overall prevalence specific to Australia was 30.4% (95% CI: 29.0–31.7%) [29]. Studies of older populations indicate that MASLD prevalence either remains stable or decreases compared with middle-aged groups. This pattern may reflect survivorship bias: individuals healthy enough to participate in prospective community-based studies at age ≥ 85 years—without prior major cardiovascular events or significant cognitive/physical dysfunction—are less likely to have metabolic comorbidities, including MASLD. A substantial Korean population demonstrated that the reduced prevalence of MASLD in the elderly cohort compared to the general one highlights the necessity of identifying risk factors specific to age. It has been demonstrated that carrying excess weight or being obese is a significant predictor of the development of MASLD and progressive fibrosis. Furthermore, additional cardiometabolic risk factors being present resulted in a heightened risk of MASLD occurrence and advanced fibrosis in the elderly population [30]. Additionally, the association between MASLD and cardiovascular disease appears less clear in older adults than in middle-aged populations. However, these findings reveal that MASLD’s current epidemiological burden is considerable and persists in rising.

### 3.2. Pathogenesis

#### 3.2.1. Parental and Prenatal Risk Factors

**Figure 1:**
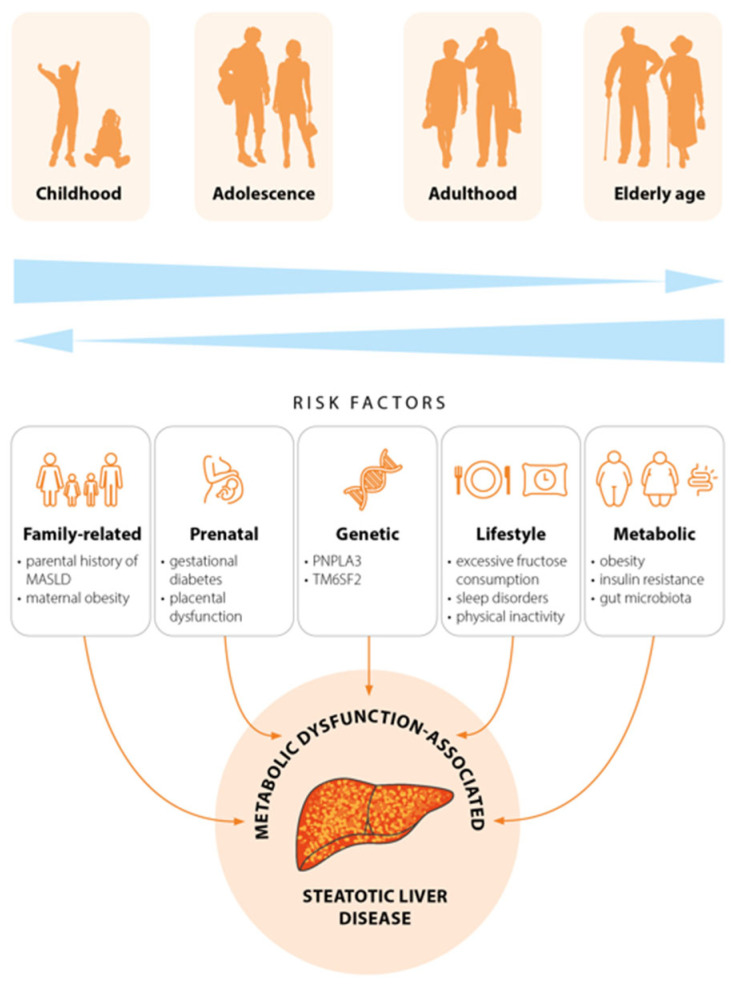
Risk factors for metabolic dysfunction-associated steatotic liver disease in different age groups.

In broad terms, MASLD stems from the correlation of metabolic, environmental, familial, genetic, and prenatal factors. The influence of these factors on MASLD development varies across different age groups (Figure 1). Much of the current understanding of MASLD pathophysiology in children stems from insights gained through adult MASLD research, owing to the scarcity of pediatric studies that comprehensively model the disease’s mechanisms in children. The “multiple hit” model remains the predominant framework explaining the pathophysiology of both pediatric and adult MASLD. The evidence confirms that hypercaloric diets (particularly those rich in carbohydrates), genetic predisposition, insulin resistance, gut dysbiosis, and sedentary lifestyle drive hepatic fat accumulation, steatohepatitis, and fibrosis [30]. Maternal metabolic status during pregnancy has enduring effects on offspring, positioning obesity and gestational diabetes as the most significant perinatal risk factors for MASLD development in childhood. These conditions have been demonstrated to disrupt maternal–fetal nutrient balance, fostering a pro-inflammatory, lipid-toxic intrauterine environment [31]. Preclinical data support a recent hypothesis that early-life adaptation to maternal obesity may impact the risk of MAFLD in offspring. This is confirmed by the study results that showed a higher prevalence of maternal obesity among offspring with MAFLD versus controls (19.3% vs. 8.4%), with a notable dose–response relationship with maternal BMI. The offspring of mothers with obesity faced a 3-fold higher MAFLD risk than those of mothers with normal weight [32]. Another study of children with obesity showed that 54.4% of those with MAFLD had at least one obese parent [33]. Interestingly, birth weight, indirectly influenced by maternal metabolic status, further modulates MASLD risk. Both low and high birth weight are associated with elevated disease risk. A study by Newton et al. [34] indicated that a high body weight in children with MASLD was associated with a significantly higher odds ratio for metabolic dysfunction-associated steatohepatitis (MASH) and liver steatosis compared to children with normal birth weight. The same study shows that a low body weight was associated with a markedly increased risk of advanced fibrosis [34].

#### 3.2.2. Genetic Risk Factors

Previous epidemiological studies indicate that MASLD has a heritable component. The development and progression of MASLD is influenced by numerous single nucleotide polymorphisms (SNPs), irrespective of age. The most extensively studied one is PNPLA3 (encoding patatin-like phospholipase domain-containing 3), particularly the rs738409 polymorphism. This variant strongly correlates with increased hepatic triglycerides [35]. Variants in GCKR (encoding glucokinase regulatory protein) and TM6SF2 (encoding transmembrane 6 superfamily member 2) also strongly influence the pathogenesis of MASLD. The TM6SF2 rs58542926 variant correlates with an elevated accumulation of hepatic fat and limited export of very-low-density lipoprotein, possibly providing protection against cardiovascular disease while increasing the risk of fibrosis [36]. What is more, the GCKR rs1260326 variant exacerbates de novo instances of liver inflammation and lipogenesis [37].

#### 3.2.3. Environmental and Metabolic Risk Factors

Environmental and metabolic risk factors contribute substantially to MASLD pathogenesis and exert a primary influence across both pediatric and adult populations. MASLD exhibits a strong linkage with metabolic dysregulation, which frequently arises from poor lifestyle choices and dietary patterns. Robust evidence demonstrates that an elevated consumption of sugar-sweetened foods promotes obesity throughout the lifespan, while serving as a key driver in MASLD development. Research in adults reveals that a high intake of sugar-sweetened beverages correlates not only with obesity but also with greater hepatic fat deposition [38]. According to the Hohenheim fructose intervention research, overweight children with MASLD ingested a notably higher number of sweetened beverages than overweight children without MASLD [39].

Metabolic disorders are the main factors in the pathogenesis of MASLD in all ages. The enlargement of adipose tissue, associated with obesity, results in its functional impairment. Dysfunctional adipose tissue secretes various bioactive compounds, including reactive oxygen species, free fatty acids, and pro-inflammatory cytokines (such as leptin, IL-6, and TNF-α), which interfere with insulin signaling pathways and ultimately induce insulin resistance [40]. The ensuing insulin resistance disrupts the homeostatic regulation of multiple glucose and lipid parameters due to persistent hyperinsulinemia. This metabolic disequilibrium contributes to the onset of T2DM and promotes de novo lipogenesis, thereby exacerbating dyslipidemia [41]. In addition, the pro-inflammatory mediators derived from visceral adipose tissue foster a chronic low-grade systemic inflammatory state, which further compromises insulin signaling [42]. The elevated influx of free fatty acids into the liver results in hepatic lipid overload and the excessive storage of lipids within hepatocytes. The accumulation of these lipids induces lipotoxicity—a cytotoxic condition caused by lipid-derived metabolites such as lipophosphatidylcholine, diacylglycerol, and ceramides. These metabolites intensify oxidative stress and elicit inflammatory responses within hepatocytes, leading to cellular dysfunction and the disruption of essential hepatic physiological processes. As a result, impaired hepatocytes lose their capacity to sustain normal lipid metabolic functions [43,44]. Thus, hepatic dysfunction aggravates disturbances in lipid metabolism and drives the development of dyslipidemia with systemic metabolic repercussions. Various metabolic factors dominate the pathogenesis of MASLD across different age groups. A longitudinal retrospective cohort study was conducted over a period of four years, with 10,240 healthy individuals enrolled. The study collected and analyzed baseline and dynamic alterations in metabolism and hepatic steatosis, determined using ultrasound imaging. The participants were divided into the following age groups: 20–34, 35–49, 50–64, and over 65 years [45]. In the younger study population, an elevated BMI accurately predicted MASLD occurrence, indicating that weight control represents the primary preventive strategy. For people aged 35–49 years, the AUC for combined baseline and dynamic BMI changes in predicting MASLD reached 0.79, indicating that prevention strategies should address both current weight and its longitudinal trajectory. In people aged 50–64 years, the AUC of baseline BMI for predicting MASLD was 0.74, and the prediction accuracy of baseline high density lipoprotein was low (AUC = 0.62), confirming weight management as the primary preventive target. For elderly people, the combination of baseline systolic blood pressure, uric acid serum level, and increases in triglyceride serum level could predict MASLD onset (AUC = 0.89). This suggests that metabolic disease treatment in the elderly is fundamental for the prevention of MASLD [45]. These findings demonstrate that MASLD risk factors vary according to population, and that targeted measures ought to be implemented to effectively limit MASLD occurrence.

#### 3.2.4. Hormonal Risk Factors

The prevalence and progression of MASLD and MASH vary with age and exhibit sex differences. A higher MASLD prevalence has been reported in men during their reproductive years than in women. The condition frequently transitions into more serious stages, such as cirrhosis, MASH, and hepatocellular carcinoma [46,47,48]. Higher levels of liver steatosis are also displayed in men in comparison to premenopausal women. However, MASLD prevalence in women becomes similar to that in men after the age of 50 [49]. This transition resulted in the collective European Association for the Study of the Liver (EASL)—European Association for the Study of Diabetes (EASD)—European Association for the Study of Obesity (EASO) recommendations on MASLD to highlight that patients with numerous cardiometabolic risk factors, postmenopausal women, and men over 50 face an increased risk for cirrhosis, progressive fibrosis, and related complications [6]. The influence of hormonal disorders is already visible in girls. A higher MASLD occurrence has been observed in patients with normal BMI and polycystic ovary syndrome. Elevated levels of androgen and hyperinsulinemia are suggested to lower the production of adiponectin and debilitate lipid metabolism, resulting in hepatic steatosis. A low level of adiponectin also tends to reduce the production of hepatic sex hormone-binding globulin, further worsening hyperandrogenism and insulin resistance [50,51]. A meta-analysis shows that, after menopause, women are at a 2.4-fold higher risk of MASLD [52]. It was also observed that the levels of estradiol were lower in premenopausal women diagnosed with MASLD than in those without MASLD. The importance of estrogens for the metabolism of lipids is relatively well-established. They limit de novo lipolysis in adipocytes and lipogenesis in the liver, and, in contrast, facilitate the beta-oxidation of fatty acids in the skeletal muscles and liver. Moreover, estrogens have an impact on the distribution of adipose tissue. In premenopausal women, it is predominantly deposited subcutaneously, limiting lipolysis and strengthening insulin sensitivity. In men and postmenopausal women, visceral fat accumulation mainly increases the risks of insulin resistance and inflammation. It is likely that subcutaneous fat influences MASLD development; however, people with obesity appear to have more inflammatory cytokines produced by macrophages in the visceral adipose tissue [53,54,55]. Data on the influence of hormone replacement therapy (HRT) on MASLD occurrence are inconsistent, which is likely due to the complex and multifactorial impact of HRT on MASLD pathogenesis. It was observed that postmenopausal women administered HRT showed a decreased prevalence of metabolic syndrome and MASLD [56,57]. Also, the influence of male sex hormones was noted in MASLD among women. MASLD progresses at low testosterone levels in men, in comparison to increased levels in women [58]. This relation was further specified in the study of Park et al. [59], who pointed out that the elevated risk of MASLD development related only to premenopausal women who were at a regular range of testosterone levels, although closer to its higher end [59]. Other research stated that high testosterone levels in this study population also led to more extensive fibrosis and an elevated MASH risk [60]. This correlation appeared to be stronger the lower the age was, and it was observed even after a cohort of women with polycystic ovary syndrome was excluded [61].

#### 3.2.5. Gut Microbiota Dysbiosis

According to a systematic review and meta-analysis incorporating new data, patients with MASLD, compared to those without it, have a similar amount of Parabacteroides, Dorea, Bifidobacterium, Roseburia, Bacteroides, Blautia, Lactobacillus, and Clostridium. Yet they have more Streptococcus, Escherichia, and Prevotella, and lower levels of Ruminococcus, Coprococcus, and Faecalibacterium [62]. The gut–liver axis is very important in hepatic steatosis development. Intestinal permeability is increased by dysbiosis due to bacterial endotoxin bloodstream ingression, and, as a result, the accumulation of fat and liver inflammation can be observed. High intestinal permeability and low levels of choline cause intestinal failure, celiac disease, and prolonged parenteral nutrition to contribute to the elevated risk of hepatic steatosis [63]. Interestingly, gut microbiome changes may affect the genes responsible for lipogenesis within the liver [64]. Gut dysbiosis in children with MASLD, compared to those without it, indicates an increase in absorbed carbohydrate oxidation [40]. Both children with MASLD and those with MASH were observed to show a marked decrease in microbial α-diversity, determined by limiting the variety of microbial taxa with the simultaneous retention of metabolic patterns. It was also reported that β-diversity, i.e., the dissimilarity between pairs of microbiomes, appeared to be more pronounced.

### 3.3. Lean MASLD

Despite overweight and obesity being well-known risk factors for MASLD, the condition may be observed also in individuals with normal body weight. It is then called lean MASLD. According to 53 studies, including 249,544 healthy people and 65,029 MASLD patients, lean MASLD occurrence in the general population was at a level of 11.2%. In the MASLD study cohort, 25.3% of lean MASLD was noted [65]. In different researched groups, the proportion of patients with lean MASLD was around 10–20%. Among 3419 patients included in The Polish Gallstone Surgery Registry, MASLD was diagnosed in 24.2% of individuals, and lean MASLD in 11.3% of patients with MASLD [66]. Prediabetes conditions or T2DM (OR 9.26, CI 4.42–19.38, *p* < 0.0001), atherogenic dyslipidaemia (OR 94.16, CI 45.54–197.71, *p* < 0.0001), and hypertension (OR 2.97, CI 1.38–6.41, *p* = 0.0054) were considered independent predictors of lean MASLD. Up-to-date studies show differences in age and sex pointing at the older male patients’ group as the one at the highest MASLD risk [67].

Expert research states that the lean MASLD group ought to include non-Asian patients with MASLD and BMI < 25 kg/m^2^ and Asian ones with BMI < 23 kg/m^2^ and MASLD. No sufficient data concerning children in terms of lean MASLD are available. It is advised to routinely assess lean MASLD patients for such comorbidities as hypertension, type T2DM, and dyslipidaemia [68]. Their liver steatosis pathogenesis is not clear, and, in addition to the recognized metabolic dysfunction, fibroblast growth factor-19, elevated secondary bile acid serum levels and gut microbiota impairments have been connected with lean MASLD development [69].

Lean MASLD individuals are more prone to a worse long-term prognosis compared with non-MASLD patients and overweight or obese patients. A seeming paradox of increased liver-related morbidity and death in subjects with regular weight and less pronounced initial liver impairment may be observed. Thus, it is necessary for further studies to research this clinically relevant paradox in detail. A thorough knowledge of the condition’s pathophysiology will contribute to addressing the matter, and is better than depending solely on BMI indicators.

### 3.4. Diagnosis

Children and adults usually do not present with symptoms of MALSD, and it is detected incidentally. Clinical manifestations, when present, are non-characteristic and may include hepatomegaly or mild right-upper-quadrant abdominal discomfort.

In June 2023, experts from international societies published a consensus recommending the division of steatotic liver disease based on etiology [5]. Both in children and adults, MASLD is diagnosed when there is evidence of hepatic fat accumulation demonstrated by histology, imaging, or biochemical markers, and at least one of the following five cardiometabolic conditions: overweight, obesity, or abdominal obesity; prediabetes or T2DM; hypertension; hypertriglyceridemia; or low high-density lipoprotein cholesterol [5]. Diagnostic criteria for these conditions differ between children and adults (Table 1).

It should be emphasized that MASLD can be concurrent with toxic or drug-induced liver steatosis. A mixed etiology of liver steatosis is not rare. It is most often encountered in populations of adult and elderly people—due to polypharmacy. Table S1 in the Supplementary Materials contains a list of active substances with established hepatotoxic potential.

The main implication for practitioners arising from the recent consensus is that MASLD has replaced the previous NAFLD terminology and is defined as hepatic steatosis ≥ 5% with no hepatocellular injury (i.e., without fibrosis, inflammation, or hepatocyte ballooning) [70]. The former NASH nomenclature (non-alcoholic steatohepatitis) has been replaced with MASH and is currently determined as hepatic steatosis ≥ 5% with or without fibrosis, with ballooning of hepatocytes (inflammation with hepatocyte injury and death) [5,70].

Redefining NAFLD terminology holds a greater urgency in pediatrics than in adults, given the atypical role of alcohol due to the near-universal legal restrictions on consumption for those under 18. In children exhibiting steatosis but lacking typical cardiometabolic risk factors, alternative etiologies must also be excluded, including parenteral nutrition, inborn errors of metabolism, lipodystrophy, hepatitis C, and steatogenic drugs such as valproate. When cardiometabolic risk factors occur alone or alongside other contributors, multifactorial MASLD etiologies must be considered [5].

The early diagnosis of MASLD is critical for mitigating disease progression and preventing long-term complications. Given epidemiological data confirming the disease’s high prevalence, active screening should begin in the youngest at-risk individuals. The American Association for the Study of Liver Diseases recently issued a practice statement recommending targeted screening in high-risk children [71]. It is strongly recommended for the overweight and obese pediatric population ≥ 10 years of age with a family history of MASLD or additional risk factors of cardiometabolic disorders. Serum alanine aminotransferase (ALT) is the principal proposed screening modality, as elastography or ultrasound are costly and not readily available imaging tools [71]. Both children and adults may develop fatty liver disease with normal ALT levels [72]. It should be emphasized that ALT has a low sensitivity, especially in early-stage steatosis or fibrosis. On the other hand, in elderly patients with long-standing MASLD, ALT activity may be normal due to hepatocyte exhaustion and the replacement of steatosis by fibrosis processes. In a study evaluating young adults, adults, and elderly individuals, Lin et al. [45] demonstrated that liver enzyme activity decreased with age in the course of MASLD. Nevertheless, ALT lacks optimal specificity and sensitivity, particularly for initial fibrosis and steatosis.

According to the majority of current clinical guidelines, abdominal ultrasound should be conducted in patients with increased ALT screening levels [6,46]. This recommendation applies universally across all age groups, and, although ultrasound is not always accessible, its cost-effectiveness has been well-established. Characteristic ultrasound findings indicative of hepatic steatosis encompass the obscured visualization of intrahepatic vasculature, enhanced hepatic echogenicity, liver parenchyma, and diaphragm, together with a hypoechoic renal cortex compared to hepatic tissue. Nevertheless, ultrasound sensitivity and interpretive reliability diminish in significantly obese patients and pediatric population due to an increased thickness of adipose tissue. The sensitivity for the recognition of mild steatosis has been reduced in standard ultrasounds. The results of the examination highly depend on the operator, and image quality and diagnostic precision decrease as the subcutaneous adipose tissue thickens. Correspondingly, transient elastography may provide inaccurate measurements and a limited reproducibility due to technical limitations. These restrictions are likely to result in a conceivably delayed diagnosis, underrating or giving a flawed assessment of fibrosis and steatosis stages, veiling the progression of diseases, or influencing follow-up and referral procedures. Hence, the interpretation of non-invasive assessments in severely obese children should be performed with caution and linked to biochemical, clinical and long-term data in the process of disease management consideration. Additionally, it should be mentioned that ultrasound detects steatosis only when >30% of hepatocytes exhibit lipid accumulation.

**Figure 2:**
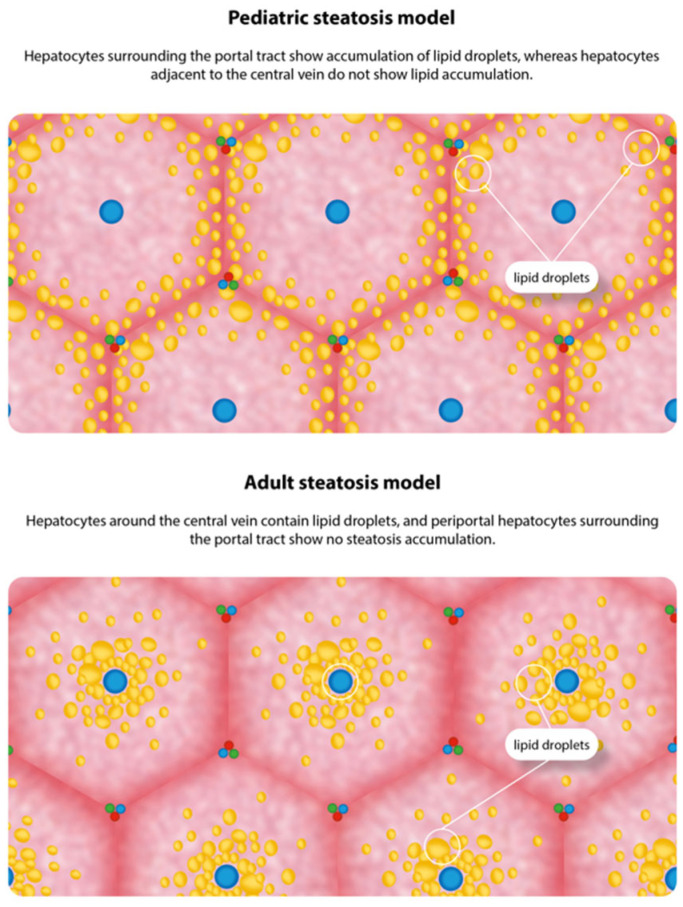
Histopathological features of steatosis in pediatric and adult metabolic dysfunction-associated steatotic liver disease.

Despite liver biopsy being the standard procedure for liver fibrosis identification, the emergence of non-invasive methods has revolutionized clinical practice—except in pediatric populations. Notably, definitive MASH diagnosis in children lacks validated non-invasive biomarkers and necessitates liver biopsy. Histologically, zone 3 lobular inflammation with hepatocyte ballooning characterizes adult MASLD, whereas pediatric cases more commonly exhibit portal inflammation and steatosis in zones 1 or 3. When fibrosis occurs, pediatric MASLD demonstrates portal/periportal distribution, contrasting with the pericellular pattern typical of adults [73]. The etiology underlying these distinctive pediatric histological features remains undetermined. Figure 2 shows the main histological differences between MASLD in children and adults. In adult patients, including the elderly, non-invasive tools—including radiology-based tests such as magnetic resonance elastography, vibration-controlled transient elastography, simple laboratory-based scores, and proprietary direct-fibrosis laboratory tests—should be utilized to diagnose liver fibrosis [74].

### 3.5. Clinical Implication

MASLD is a clinically heterogeneous condition. A subset of patients exhibits MASH features that frequently progress to fibrosis, cirrhosis, and hepatocellular carcinoma. However, hepatic complications among individuals with MASLD are relatively uncommon, in contrast to metabolic and cardiovascular complications [75].

The absence of longitudinal natural history research in pediatric MASLD has resulted in a substantial knowledge gap regarding the temporal evolution of the disease. This lack of longitudinal data limits the ability to delineate predictors of advanced disease, highlighting the critical need for robust, long-term studies in this population. The existing data indicate that unaddressed metabolic dysfunction during childhood is associated with progressive hepatic injury, inflammation, and fibrotic remodeling, mirroring patterns described in adult cohorts. Importantly, MASLD remains potentially reversible prior to the development of advanced fibrosis, emphasizing the importance of initiating therapeutic interventions early in the disease course. In the absence of timely management, there is a significant likelihood of progression to MASH accompanied by fibrosis, potentially evolving into cirrhosis in late adolescence or adult life. Two double-blind, randomized clinical trials monitored children with MASLD who received a placebo along with standard lifestyle recommendations. Follow-up liver assessments after a median period of 1.6 years showed that 29% of those with borderline or definite MASH at baseline improved to no MASH, while 18% of children with either fatty liver or borderline MASH experienced progression to definite MASH. The resolution of MASLD occurred in 2.4% of participants, all of whom initially presented with steatosis but not NASH. Fibrosis regressed in 34% of cases but worsened in 23%. Factors predicting disease progression over time included rising levels of ALT, hemoglobin A1c, and gamma-glutamyl transpeptidase (GGTP), as well as the onset of T2DM. These markers may assist in identifying patients requiring more aggressive treatment or repeat liver biopsy. Notably, 7% of the cohort developed new-onset T2DM in two years—an occurrence nearly 300-fold higher than that observed in the general pediatric population [76]. According to data from the United Network for Organ Sharing, MASH or cryptogenic cirrhosis accounted for 7% of liver transplants among adolescents and young adults [52]. Similarly, a Swedish cohort study conducted between 1966 and 2017 involving 718 pediatric and young adult patients with biopsy-confirmed MASLD demonstrated significantly increased risks of overall, liver-related, cancer-related, and cardiometabolic mortality compared with matched controls [77].

MASLD in adults is not related to hepatic endpoints in the general population; however, it may be associated with an elevated risk of non-liver complications. The more cardiometabolic risk factors, the higher the risk. Some researchers have pointed to increased mortality due to cardiovascular diseases [78,79], but others have observed it only in populations with biopsy-proven MASH [80] or with other conditions, e.g., T2DM [81], not in the general population. In summary, MASLD patients are more prone to chronic kidney disease (HR 1.43), coronary heart disease (odds ratio [OR] 1.33), heart failure (OR 1.5), non-fatal cardiovascular disease (HR 1.40), obstructive sleep apnea (OSA, HR 2.22), and T2DM (HR 2.19) [6]. In the general population, a link between MASLD and the more frequent occurrence of hepatocellular carcinoma and particular extrahepatic cancers, mainly gastrointestinal and thyroid [58], can be observed. However, no such connection has been noted in terms of MASLD and elevated general cancer-related mortality [82].

The older MASLD populations are recommended to estimate cardiovascular risk with the use of a suitable risk calculator [6]. Nevertheless, it has not been confirmed whether MASLD itself remains an independent risk for cardiovascular conditions in the elderly or if MASLD-specific treatment can prevent or restrict cardiovascular-related mortality or atherosclerotic cardiovascular disorders. A recent study has shown an overall reduction in life expectancy among patients with NAFLD; however, this risk appears to diminish with increasing age beyond 70 years, and especially after 80 years [83]. In contrast, a study of hepatic steatosis in an older community cohort showed no significant association between steatotic liver disease and mortality [84]. In another study, NAFLD was associated with all-cause and cardiovascular mortality in patients aged 60–74 years, but that association was not observed in those ≥75 years of age [27]. This reduction in mortality may be partly explained by survivorship bias; however, it may also reflect that MASLD presents differently in older adults compared with middle-aged individuals.

### 3.6. Treatment

#### 3.6.1. Non-Pharmacological Treatment

The recommendation for patients to alter their style of life continues to be the cornerstone of MASLD treatment in different age populations. It is the primary guidance in all documents concerning the management of MASLD cohorts [6,49,70]. Multiple clinical studies point out that an appropriate diet accompanied with physical activity substantially mitigates liver steatosis, proven by laboratory tests and/or imaging, and reduces body weight. The guidelines concerning the management of children with MASLD are not as numerous as those for adults. In 2017, a document was published that precisely outlined the principles of non-pharmacological treatment of MASLD in children: a well-balanced diet, regular engagement in daily moderate-to-vigorous physical activity, avoidance of sugar-sweetened beverages, and limiting screen time to less than two hours per day [85]. In contrast, the current American Association for the Study of Liver Diseases recommendations emphasize that the optimal type of diet and the intensity and duration of physical activity associated with the best therapeutic outcomes in children with MASLD and obesity remain not fully clarified [71]. For children with MASLD, it is recommended to control the total energy intake and limit the consumption of free sugars and saturated fats. In a randomized controlled trial focused on adolescent boys with MASLD, an eight-week diet with a low amount of free sugar considerably enhanced liver steatosis compared to the regular, non-reduction diet [86]. Evidence from multiple countries shows how important individual behaviors, i.e., regular physical activity and a healthy diet, are. It also emphasizes the relevance of environmental factors, such as family and school determinants. A study encompassing an obese pediatric population introduced a thorough lifestyle strategy. The follow-up findings revealed that the intervention cohort significantly reduced BMI, contrary to controls. It also stabilized obesity occurrence at 27%, while the control group reached the level of only 5.6% [87].

In adults with MASLD, numerous clinical trials have consistently shown that weight loss attained through caloric restriction, with or without concurrent increases in physical activity, results in significant improvements in MASLD-related biomarkers such as hepatic enzymes, MASH, steatosis, and fibrosis [6]. The magnitude of these improvements exhibits a dose-dependent relationship with the degree of weight reduction. Nevertheless, the current evidence remains inadequate to substantiate a beneficial effect of lifestyle-induced weight loss on severe cirrhosis or fibrosis, mainly owing to the scarce number of patients with advanced fibrosis in most clinical studies and the lack of thorough subgroup analyses [6]. To improve MASLD adults’ liver injuries, assessed in histological examinations or with the use of non-invasive modalities, it is highly recommended to reduce the intake of ultra-processed foods containing sugars and saturated fats as well as sugar-sweetened beverages, and enhance diet quality by adopting a Mediterranean-like dietary pattern [6]. It is noteworthy that this kind of diet appears to add value to cardiometabolic health and the reduction of hepatic lipids and seems to be easy to follow longitudinally by patients with MASLD. It features high intakes of fruits, vegetables, nuts and seeds, fish, seafood, legumes, olive oil, and whole grains. Thus, it supports a decreased consumption of ultra-processed foods, red meat, saturated fats, refined carbohydrates and sugars, which all contribute to the growing risk of MASLD [88]. Although cardiometabolic benefits have been well-established, this does not apply to the influence of exercise and physical activity benefits on histological results, fibrosis, the non-invasive assessment of hepatic damage, and liver-specific clinical events. Yet adult patients with MASLD ought to receive personalized exercise recommendations based on their abilities and preferences (ideally 75 min/week vigorous-intensity or >150 min/week moderate activity) [6].

In the elderly population with MASLD, multiple obstacles, such as motivational, financial, and functional difficulties, low food appeal, and psychosocial barriers, appear frequently while implementing dietary improvements and lifestyle alterations [49]. It should be highlighted that there are significant additional comorbidities in older individuals with MASLD compared to middle-aged patients. Given these considerations, prioritizing weight reduction through dietary restriction alone may pose increased risks in this population. Alternatively, emphasizing improvements in overall dietary quality—such as adopting a Mediterranean-style eating pattern—could represent a more appropriate therapeutic aim; however, both strategies present practical challenges to implementation. Furthermore, elderly lean MASLD patients scarcely benefit from a considerable loss of weight. Some data identify substantial weight loss as the reason for the elevated mortality risk [89]; however, it is not clear what implications this population specifically may bear. Older adults with MASLD are at a higher risk of being pre-frail or frail and of developing lasting physical disability, while substantial weight loss itself constitutes an independent risk factor for frailty development [90]. Nevertheless, the available evidence indicates that certain obese older individuals may experience functional gains from weight reduction, particularly when it is accompanied by structured exercise [91]. Understanding the optimal habitual management strategies for older persons with MASLD requires further study.

It is possible to reestablish the balance of gut microbiota through probiotics—health-promoting microbes, postbiotics—probiotic byproducts, or prebiotics—non-digestible carbohydrates [92]. A meta-analysis based on 25 investigations into prebiotics applications in obesity has confirmed their considerable influence on adipose accumulation and weight gain [93]. Nevertheless, it seems they have little, arguably a preliminary and partial, effect on specific patients with MASLD. In contrast, evidence from both animal models and human trials supports probiotics’ role in MASLD and reveals decreased hepatic inflammation, the efficacious modulation of gut microbiota, and reduced fat deposition in liver. In particular, varying effects arise from specific single-strain or multi-strain probiotic formulations. Fecal microbiota transplantation (FMT) entails transferring donor-derived, processed fecal bacteria to the recipient’s intestines to successfully alter gut dysbiosis, and favorably modulate animal and human metabolism. The analysis of FMT trials in MASLD patients showed that, after undergoing a 6-week intervention of FMT using microbiota from lean, healthy providers, recipients seemed to have an enhanced liver insulin sensitivity, and their butyrate-producing bacteria abundance grew. Butyrate considerably changes insulin resistance and mitigates endotoxic bacterial particle translocation [94]. Notably, FMT normalizes the microbiota–liver axis in hepatic steatosis patients, with a significant recovery of impaired intestinal barrier function observed six weeks after allogeneic FMT [95]. The FMT investigations reveal auspicious evidence connected with patients’ microbiota rebalancing and enhanced intestinal barrier integrity, which leads to MASLD improvement.

#### 3.6.2. Pharmacological Treatment

Currently, pharmacological treatment options for pediatric MASLD are extremely limited. No medications are specifically approved for MASLD in children, and the available agents provide only minor advantages in selected populations. Glucagon-like peptide-1 (GLP-1) receptor agonists are most prospective for pediatric MASLD populations due to their considerable effectiveness in the treatment of obesity and related metabolic disorders [71]. The randomized, placebo-controlled study, which encompassed children with MASLD, revealed that, after a 96-week treatment, metformin and vitamin E maintained reduced ALT levels relative to a placebo [96]. Currently, the sole recommended pharmacologic approach for pediatric MASLD is aimed at comorbidities such as arterial hypertension, dyslipidemia, and T2DM (with GLP-1 receptor agonists approved for children aged ≥10 years) [71]; however, it should be applied with caution.

According to the current statement from the European Association for the Study of Obesity, European Association for the Study of Diabetes, and European Association for the Study of the Liver, nutraceuticals cannot be recommended in adults with MASLD, as there is insufficient evidence regarding their effectiveness or safety in reducing histologically/non-invasively assessed liver damage/fibrosis or liver-related outcomes [6]. Furthermore, adults with non-cirrhotic MASH and advanced hepatic fibrosis ought to receive resmetirom as a MASH-specific treatment due to its histological improvement in steatohepatitis and fibrosis and acceptable safety and tolerability. What is more, treatment with resmetirom may be considered for individuals with MASLD who are non-cirrhotic and have documentation of either advanced fibrosis, at-risk steatohepatitis with significant fibrosis, or an elevated risk of adverse liver-related outcomes [6]. It should be emphasized that, in MASLD patients, the treatment of comorbidities is important for MASLD clinical course and liver-related prognosis [6]. Hypoglycemic agents, particularly inhibitors of sodium glucose cotransporter-2 (SGLT2) and glucagon-like peptide-1 (GLP-1) analogs, are observed to diminish inflammation, improve insulin sensitivity and metabolic parameters and reduce hepatic steatosis. Tirzepatide, which is a dual agonist of GLP-1/glucose-dependent insulinotropic polypeptide receptors, has demonstrated a significant effect in reducing the degree of hepatic steatosis and improving metabolic parameters accompanied by effective weight reduction [97]. Therefore, the mentioned drugs should be chosen for patients with MASLD and T2DM.

In older adults with MASLD, the consideration of polypharmacy and the competing risks associated with aging, along with the careful selection of adjunctive therapies for coexisting conditions, represent an important yet insufficiently explored area of research. Currently, no data are available regarding the use of resmetirom in this population. This gap is noteworthy given the established associations between aging and thyroid dysfunction, the influence of thyroid status on cognitive performance, and the tendency toward increased thyroid hormone resistance in older individuals [6]. It ought to be established whether the gastrointestinal adverse effects of resmetirom could contribute to unfavorable dietary changes in older patients with MASLD. Moreover, as older individuals are frequently treated with multiple medications, the potential effect of polypragmasy on the resmetirom and other drugs’ efficacy has not been adequately studied [6]. It should be noted that, in older patients, the side effects of drugs may be more frequent and severe than in younger populations. Pharmaceuticals such as tirzepatide, GLP-1 analogs or SGLT2 inhibitors administered in the elderly with T2DM and MASLD may increase the risk of hypoglycemia and all its unfavorable consequences, gastrointestinal adverse reactions, nausea and vomiting. Additionally, SGLT2 inhibitors and pioglitazone may induce bone loss, which is particularly dangerous in this population.

## 4. Aging-Related Differences in Clinical Aspects of MASLD

The consequences of aging cannot be attributed solely to disease accumulation or the cumulative impact of risk factors. Chronological age, in fact, is an imprecise indicator of biological aging, which varies considerably both among individuals and across organ systems within the same person. This underscores the necessity for targeted, aging-specific research on prevalent conditions such as MASLD, since extrapolating findings from studies conducted in younger adults carries substantial limitations. However, some aspects of MASLD across different age groups are well established and hold important clinical relevance. Firstly, the prevalence of MASLD is high across all age groups, with a steady increase in incidence projected over time. Moreover, MASLD is being diagnosed in progressively younger patients. In the context of serious metabolic and cardiovascular comorbidities that accompany MASLD and worsen prognosis, it should be emphasized that implementing diagnostic protocols aimed at the early identification of affected individuals, including children, is essential. The general MASLD burden may be reduced by applying age-specific metabolic prevention methods. Secondly, the risk factors for MASLD differ across age groups. In children, prenatal and familial factors predominate, whereas, with increasing age, metabolic determinants play a greater role. For years, excessive body weight has remained the principal risk factor for the development of MASLD; therefore, screening for MASLD in individuals who are overweight or obese is recommended at all ages. Thirdly, as obesity represents a principal determinant of MASLD, the considerable health and economic gains achieved through obesity-targeted interventions imply that a comparable investment in MASLD prevention—especially by implementing lifestyle modifications early in life—could produce substantial benefits by reducing the progression of subsequent metabolic complications. However, these assumptions still require forward-looking analysis and validation in young and elderly MASLD populations. In summary, age-dependent differences in the epidemiology, clinical course, prevention, and treatment of MASLD emphasize the vital need for personalized intervention and prevention strategies to approach the disease in an effective way.

## Figures and Tables

**Table 1:** Cardiometabolic criteria for MASLD diagnosis [5].

Criteria	Children	Adults
Weight	BMI ≥ 85th percentile for age/sex (BMI Z-score ≥ +1) or WC > 95th percentile	BMI ≥ 25 kg/m^2^ (23 kg/m^2^ for Asians) or WC > 94 cm (males), >80 cm (females)
Glucose	Established diagnosis of T2DM or specific treatment for T2DM or	
	Fasting serum glucose ≥ 100 mg/dL or random serum glucose ≥ 200 mg/dL or 2 h oral glucose tolerance test ≥ 140 mg/dL or HbA1c ≥ 5.7%	Fasting serum glucose ≥ 100 mg/dL or 2 h oral glucose tolerance test ≥ 140 mg/dL or HbA1c ≥ 5.7%
Lipids	Lipid-lowering treatment or	
	Age < 10 years: triglycerides ≥ 100 mg/dL Age ≥ 10 years: triglycerides ≥ 150 mg/dL or HDL-cholesterol ≤ 40 mg/dL	Triglycerides ≥ 150 mg/dL or HDL-cholesterol ≤ 40 mg/dL (males), ≤50 mg/dL (females)
Blood pressure	Antihypertensive treatment or	
	Age < 13 years: BP ≥ 95th percentile or ≥130/80 mmHg Age ≥ 13 years: BP ≥ 130/80 mmHg	BP ≥ 130/85 mmHg

BMI—body mass index, BP—blood pressure, HbA1c—hemoglobin A1c, HDL—high-density lipoprotein, T2DM—type 2 diabetes mellitus, WC—waist circumference, Z-score—standard score.

## Data Availability

No new data were created or analyzed in this study.

## Supplementary Materials

The following supporting information can be downloaded at: https://www.mdpi.com/article/10.3390/jcm15041536/s1, Table S1. Drug-Induced Liver Injury—mechanisms and types.
