# Supplementary material for: From Childhood to Old Age: Current Knowledge and Practical Approaches to Metabolic Dysfunction-Associated Steatotic Liver Disease
Source: J Clin Med. 2026 Feb 15;15(4):1536. doi: 10.3390/jcm15041536 (PMC12942451; doi:10.3390/jcm15041536)
Supplement: Supplementary file 1 [file jcm-15-01536-s001.zip › jcm-4129696-supplementary.pdf]

Table S1. Drug-Induced Liver Injury—mechanisms and types.

Drug	Key Mechanism	Liver Injury Pattern
Acetaminophen	Induces dose-dependent oxidative stress and hepatocellular injury	Steatohepatitis / acute liver failure risk
Amiodarone	Induces mitochondrial dysfunction, resulting in phospholipid buildup and increased oxidative stress.	Steatohepatitis, fibrosis
Antiretroviral agents	Inhibit mitochondrial DNA polymerase gamma	Steatosis due to mitochondrial dysfunctions
Corticosteroids	Induce insulin resistance and promote hepatic triglyceride accumulation	Macrovesicular steatosis
Estrogen/Hormonal Agent	Modifies hepatic lipid metabolism, leading to intracellular fat deposition	Steatosis, potential progression to steatohepatitis
Methotrexate	Causes oxidative stress and mitochondrial dysfunction, resulting in altered lipid metabolism	Steatosis, potential progression to fibrosis
Nonsteroidal antiinflammatory drugs	Triggers oxidative stress with potential mitochondrial damage	Steatosis, variable severity
Tamoxifen	Modulates estrogen receptor signaling and disrupts lipid metabolism	Steatosis with possible steatohepatitis
Tetracyclines	Leads to impaired mitochondrial function with inhibition of fatty acid oxidation	Microvesicular steatosis
Valproic acid	Disrupts mitochondrial beta-oxidation, resulting in lipid accumulation	Microvesicular steatosis
